# A national-level examination of First Nations peoples’ mental health data: Predicting mental well-being from social determinants of health using the 2017 Aboriginal Peoples Survey

**DOI:** 10.3389/fpubh.2023.1073817

**Published:** 2023-03-31

**Authors:** Lydia J. Hicks, Christopher J. Mushquash, Elaine Toombs

**Affiliations:** ^1^Department of Psychology, Lakehead University, Thunder Bay, ON, Canada; ^2^Dilico Anishinabek Family Care, Thunder Bay, ON, Canada; ^3^Center for Rural and Northern Health Research, Lakehead University, Thunder Bay, ON, Canada; ^4^Thunder Bay Regional Health Sciences Center, Lakehead University, Thunder Bay, ON, Canada; ^5^Thunder Bay Regional Health Research Institute, Lakehead University, Thunder Bay, ON, Canada

**Keywords:** social determinants of health, Indigenous mental well-being, First Nations mental well-being, Aboriginal Peoples Survey, Community Well-Being Index

## Abstract

**Introduction:**

A history of colonization and assimilation have resulted in social, economic, and political disparities for Indigenous people in Canada. Decades of discriminatory policies (e.g., the Indian Act, the Residential School System) have led to numerous health and mental health inequities, which have been intergenerationally maintained. Four main social determinants of health (i.e., income, education, employment, and housing) disproportionately influence the health of Indigenous peoples. These four social determinants have also been used within the Community Well-Being (CWB) index, which assesses the socio-economic wellbeing of a community. This study sought to extend previous research by assessing how specific indicators of CWB predict self-reported mental wellbeing within First Nations populations across Canada in a national dataset with more recent data.

**Methods:**

This study utilized the 2017 Aboriginal Peoples Survey, which includes data on the social and economic conditions of First Nations people living off reserve aged 15 years and over.

**Results:**

Results from a factorial ANOVA indicated that perceptions of income security, housing satisfaction, higher education, and employment are associated with increased self-reported mental health among First Nations individuals living off-reserve.

**Discussion:**

These results support the idea that individual mental health interventions on their own are not enough; instead, broader social interventions aimed at addressing inequities in various social determinants of health (e.g., housing first initiatives) are needed to better support individual wellbeing.

## 1. Introduction

Indigenous^1^ populations in Canada experience a disproportionate burden of ill health and social challenges than non-Indigenous populations in Canada (1). Health *disparities* are indicators of a relative disproportionate burden of health conditions on a particular population (1). Widespread disparities in morbidity, mortality, and chronic diseases (e.g., diabetes, arthritis, and high blood pressure) exist between Indigenous and non-Indigenous populations (1, 2). Disparities also exist in social challenges (sometimes called diseases of despair), with higher rates of suicide and self-injury (2), and family violence, sexual abuse, and addiction for Indigenous populations in Canada (1).

Health *inequities* are the underlying mechanisms of such health disparities, which are rooted in social, economic, cultural, and political imbalances (1). Reading and Wien’s (3) *Social Determinants Model of Aboriginal Health* was put forward to better understand how health disparities are related to various social determinants of health. This model examines social determinants of health for Indigenous populations within the socio-political context that they were established in. Contrary to other social determinants of health frameworks, Reading and Wein embrace a holistic model of health, which highlights the interrelatedness of physical, spiritual, emotional, and mental dimensions and contrasts with a “silo” approach to prevention and treatment that is commonly found in non-Indigenous health literature (3). This model demonstrates how inequities in social determinants of health can lead to additional burdens of health problems over the life course, but also how health problems themselves can perpetuate conditions (or determinants) that further impact health.

### 1.1. Distal determinants of health

Distal determinants of health include the historic, political, social, and economic contexts that influence a wide range of health vulnerabilities, capacities, and behaviors (3). While distal determinants are noted to have the most wide-reaching influence on health, they are often the most difficult to intervene on because they represent the context in which intermediate (e.g., community infrastructure and resources, social systems) and proximal (e.g., direct impacts on health, such as health behaviors and physical environments) determinants exist (3).

Among studies of First Nation health, colonialism is a commonly cited distal determinant of health and wellbeing within these populations (3, 4). Such practices include the use of colonial institutions (e.g., the Canadian government) and systems (e.g., residential schools) that were imposed upon Indigenous peoples with culture and lifestyles disrupted in various ways (e.g., the seizure of land, banning of languages, persecution of spiritual practices, and disruption of cultural values and identity (5)). Experiences of colonization have produced political, economic, and social inequalities *via* the construction of unfavorable intermediate and proximal determinants (3), which are often beyond the individual or community’s control or means to intervene upon (4).

Additional distal determinants include racism and social exclusion, as well as self-determination (3). Constructed social stratifications along “racial” lines have subsequently led to inequitable distribution of resources, power, control, and freedom. Unequal distribution of such resources can increase exposure to health damaging intermediate and proximal determinants, which in turn increases vulnerability to illness and creates further barriers for addressing health issues. Self-determination similarly influences intermediate and proximal determinants (e.g., education, housing, and health opportunities), ensuring that Indigenous peoples participate in political decision making and have control over their lands, economies, education systems, and health systems (3). Self-determination of land claims, economic decisions, and self-governance structures have been described to be more common among First Nation communities with higher wellbeing scores (6), and self-determined treaty rights have been associated with higher community income (7).

### 1.2. Intermediate determinants of health

Intermediate determinants of health are constructed by the distal determinants and can be thought of as the source or foundation of proximal determinants (3). For Indigenous peoples in Canada, intermediate determinants include health care systems (e.g., limited culturally- and contextually-appropriate services), educational systems (e.g., underfunded programming), community infrastructure and capacities (e.g., inadequate social resources), environmental stewardship (e.g., inabilities to benefit from the profits of resource extraction), and cultural continuity (e.g., traditional intergenerational connectedness with language and spirituality). Examples of how intermediate determinants impact proximal determinants include: deleterious physical environments rooted in limited community resources; and barriers to developing health promoting behaviors due to inequitable health care (3).

### 1.3. Proximal determinants of health

Proximal determinants of health are the conditions that directly impact physical, emotional, mental, and/or spiritual health (3). These determinants include: health behaviors (e.g., misuse of alcohol, excessive smoking); physical environments (e.g., housing shortages and poor quality of existing homes); employment and income (e.g., scarce economic opportunities and community resources); education (e.g., poor literacy); and food insecurity (e.g., compromised diets when food is no longer available or of poor nutritional quality). While each of these determinants are important, education, employment, income, and housing have been highlighted often within the literature because of their inclusion in the Community Well-Being (CWB) Index of Canada (8, 9). The CWB is described in further detail below following a review of these key social determinants.

#### 1.3.1. Education

Indigenous populations are less likely to complete high school and post-secondary education than non-Indigenous populations (8, 10). The largest gaps in levels of high school completion and completion of a university degree occur between First Nations individuals living on reserve and Inuit when compared to non-Indigenous populations (10). Completing a formal education (i.e., at the high school level or above) ensures individuals learn the literacy and numeracy skills needed to participate in society and access the benefits they are entitled to (10). Post-secondary educational attainment has been associated with increased employment rates and income (11). In contrast, low rates of youth engagement in education, low literacy levels, and low numeracy levels have been listed as indicators of poverty (12). Low levels of educational attainment have also been associated with various health outcomes [e.g., type 2 diabetes prevalence (8)].

#### 1.3.2. Employment

Indigenous populations have lower employment rates than non-Indigenous populations in Canada (8, 10). This differs between Indigenous groups, with First Nations individuals living on reserve and Inuit having lower employment rates than First Nations individuals living off reserve and Métis individuals (10). Unemployment has been closely linked with greater risks of chronic diseases, cardiovascular mortalities, and mental health challenges (13–15).

#### 1.3.3. Income

Indigenous populations are more likely to have lower income and higher poverty rates than non-Indigenous populations (8, 10). The gap in median income for the working age population (aged 25–64) is largest for First Nations individuals living on reserve, whose median income is less than half of that of the non-Indigenous population. Registered First Nations individuals living off reserve and Inuit have median incomes that are about 75–80% of that of the non-Indigenous population, while Métis have a median income similar to that the non-Indigenous population (10). The relationship between income and health-related outcomes is well-established: the poorer one is, the more likely they are to experience health risks in daily life (16). Key health risks that have been found for individuals with lower income include: higher rates of smoking and reporting multiple chronic conditions, as well as lower rates of physical activity and inadequate fruit and vegetable intake (16). Individuals with lower income are also less likely to receive quality health care (e.g., reduced routine screenings, such as for cervical or colorectal cancer; reduced access to prescription medical insurance; increased visits to the emergency department (16)). In addition, individuals with lower income are more likely to have worse health outcomes, such as increased rates of hospitalization for conditions that could be managed outside the hospital (16).

#### 1.3.4. Housing

Indigenous populations are more likely to live in inappropriate housing conditions (8). In 2016, close to one-fifth (18.3%) of the Indigenous population in Canada lived in housing that was overcrowded (i.e., dwellings that have more than one person per room (10)). The percentage of overcrowding varies drastically between Indigenous groups, with crowded dwellings being less common among First Nations individuals living off reserve and Métis individuals. In contrast, crowding is more common for First Nations individuals living on reserve and Inuit (10). As well, in 2016, close to one-fifth (19.4%) of Indigenous people in Canada lived in a dwelling that needed major repairs (17). Again, First Nations individuals living on reserve and Inuit had the largest percentages of dwellings in need of major repair (10). Inappropriate housing conditions have been associated with a variety of health issues (8, 18). For example, exposure to indoor dampness and mold has been associated with respiratory conditions, exposure to excessively low indoor temperatures for long periods of time has been associated with cardiovascular diseases, exposure to dwellings with features likely to cause an accident has been associated with physical injuries, and exposure to overcrowding has been associated with the spreading of infections such as tuberculosis (18). Poor housing can also induce chronic stress with symptoms of anxiety, depression, hostility, and frustration, and exposure to lead-based paint (commonly found in poor housing) can lead to various cognitive, developmental, neurological, and behavioral effects (18).

### 1.4. The Community Well-Being Index

To better conceptualize the disparities in these proximal determinants specifically between First Nations communities and other communities in Canada, McHardy and O’Sullivan developed the Community Well-Being (CWB) Index (9). The CWB summarizes four community level indicators: education, labor force, income, and housing. Scores range from 0 to 1, (with higher scores indicating greater wellbeing in each of these domains) and provide a quantitative comparator of First Nations communities and non-Indigenous communities. Such standardization allows for examination of variability in well-being within communities to be assessed and compared and also allows for trends in well-being to be tracked over time by examining if scores are improving, declining, or remaining stable (19).

There is a striking inequity among CWB scores of First Nations and non-Indigenous Canadian communities in Canada, which continues to persist across five-year iterations of CWB analyses. While the average CWB score for First Nations communities has been steadily increasing over the 35 years that CWB scores were being indexed (i.e., starting in 1981 and analyzed in 2016), a substantial gap was found in 2016 between CWB scores of First Nations and non-Indigenous communities. That is, the average CWB score for First Nations communities was 19.1 points lower than the average for non-Indigenous communities (20). Gaps in each CWB component (i.e., education, labor force, income, and housing) have remained relatively stable across time, and even where the gaps have been documented to be narrowing (e.g., education, employment, and income), they still remain wide. Overall, these socio-economic gaps between First Nations and non-Indigenous communities are significant in size and longstanding across cycles of the CWB Index (20).

Research has examined how the CWB Index relates to reports of personal (in contrast to community) well-being. For example, Wingert and White (21) examined the relationship between First Nations communities’ CWB total scores and subjective dimensions of individual well-being. CWB total scores were calculated using community level data from the Census of Canada and subjective reports of individual well-being were assessed *via* telephone surveys. Personal well-being scores were reported from 1,274 individuals. Results indicated that those in lower CWB communities more strongly agreed to feeling sad or depressed, and to having no hope for the future. Those in higher CWB communities most strongly agreed to having control over things in their lives. As well, respondents in communities with higher CWB scores most strongly agreed that they were a good person and that they were a person of worth. The authors highlighted that these patterns fit with the expectation that higher CWB communities better support the wellbeing of residents. However, this study did not examine the specific relationships between individual CWB indicators (i.e., education, employment, income, and housing) and wellbeing variables, leaving a gap in the literature in need of further explanation. The authors also highlighted that the study may be limited by low response rates and the possible systematic differences within the sample (e.g., an overrepresentation of university-educated individuals, an underrepresentation of individuals without access to a telephone) and suggested that results should be corroborated with those from other datasets, such as the Aboriginal Peoples Survey (APS) (21).

### 1.5. Study purpose and hypotheses

The purpose of the present study is to provide a more recent investigation into how indicators from the CWB framework (i.e., education, employment, income, and housing) predict subjective mental health reports for First Nations individuals in national-level, population data from the APS 2017 survey. This is the first study to our knowledge that examines this relationship between specific indicators of CWB and subjective wellbeing *via* the 2017 APS dataset. It is hypothesized that increased access to higher levels of each the four proximal determinants of health (i.e., education, employment, income, and housing) will significantly and positively predict self-reported mental wellbeing. Age, sex, and place of residence will also be examined in this model as control variables as these variables have been found to impact mental health outcomes (e.g., younger First Nations adults are more likely to speak with a professional regarding mental health concerns than older adults (22); being female is predictive of meeting criteria for various mental disorders (23); and distress scores are higher in remote communities than in urban communities (22)).

## 2. Methods

### 2.1. Use of population-level data to explore mental well-being predictors

The 2017 APS provides an opportunity to explore the relationships between social determinants of health and self-reported mental health among various communities of Indigenous people in Canada. The 2017 APS was the fifth iteration of a cross-sectional survey of Indigenous health outcomes, sociodemographic information, social determinants of health, and other variables, for First Nations individuals living off-reserve, as well as for Métis and Inuit individuals living in Canada. This cycle was conducted between January 16, 2016 and August 15, 2017 (24). Questions in the APS 2017 were designed for and administered in a Computer Assisted Interviewing (CAI) environment, which allows for more complex questionnaire flow as well as real-time edits when logical inconsistencies between questions are detected. Computer assisted telephone interviews and computer assisted personal interviews were used for this survey (25). The 2017 APS Public Use Microdata File (PUMF) was publicly accessed online for this study. As this data file is provided to a much wider range of users than the 2017 APS analytic file (available through Statistics Canada’s Research Data Centers), the level of detail is not as fine as that of the analytic file. Actions have been taken to reduce or eliminate the risk of disclosure on the PUMF, including limiting the geographic detail available on the PUMF and limiting the amount of family and household information available on the PUMF. Additional actions to lessen risk of disclosure can be found in the APS 2017 User’s Guide to the PUMF (24).

### 2.2. Participants

Participants were selected from survey respondents of the APS (2017) aged 15 years or older. Individuals who responded, “First Nations” to the survey item “Are you First Nations, Métis, or Inuk?” were included in the present analyses. Relevant participant demographic information obtained from the APS 2017 is included in Table 1.

**Table 1:** Participant demographics and ratings of self-reported mental health.

		Survey responses		Self-reported mental health	
	Demographic	Frequency	%	*M*	*SD*
Sex	Male	3,617	44.28	2.70	1.07
	Female	4,551	55.72	2.45	1.12
Age	15–18	349	4.27	2.37	1.17
	19–24	1,918	23.48	2.45	1.14
	25–34	1,170	14.32	2.57	1.08
	35–44	1,022	12.51	2.62	1.09
	45–54	1,137	13.92	2.51	1.14
	55+	2,572	31.49	2.65	1.06
Place of Residence	Other rural area	1,602	19.61	2.66	1.07
	Other population center	2,505	30.67	2.56	1.08
	Census metropolitan area	4,061	49.72	2.52	1.13
Income	Not enough	1,904	23.31	2.03	1.17
	Enough	4,439	77.66	2.60	1.03
	More than enough	1,825	22.34	3.01	0.98
Employment	Unemployed	3,525	43.16	2.38	1.14
	Employed	4,643	56.84	2.70	1.05
Education	Grade 8 or lower	438	5.36	2.25	1.10
	Some secondary education	1,075	13.16	2.35	1.13
	Secondary school diploma	1,373	16.81	2.55	1.10
	Some postsecondary education	1,721	21.07	2.52	1.12
	Post-secondary diploma	2,662	32.59	2.62	1.08
	Bachelor’s degree or more	899	11.01	2.85	1.03
Housing	Dissatisfied or very dissatisfied	837	10.25	2.03	1.19
	Neither satisfied nor dissatisfied	170	2.08	2.05	1.01
	Satisfied	4,007	49.06	2.42	1.05
	Very satisfied	3,154	38.61	2.90	1.06

### 2.3. Variables from the 2017 APS dataset

#### 2.3.1. Demographics

Age, sex, and place of residence variables (Table 1) were obtained from the APS 2017. Age was reported in terms of the age group that the respondent belonged to as of the survey reference date (i.e., January 15th, 2017). Categories included between the ages of 15–18, 19–24, 25–34, 35–44, 45–54, and 55 and over. Sex of the respondent was collected and coded as either Male or Female. Place of residence indicated whether the person was living in a Census Metropolitan Area (CMA), other population center, or other rural area. A CMA is formed when one or more adjacent municipalities are centered on a population center (i.e., the core). A CMA has a total population of at least 100,000, of which 50,000 or more live in the core. CMAs are counted as large population centers (100,000 or more people), while other population centers include medium population centers (30,000–99,999 people) and small population centers (1,000–29,999 people). The “Other rural area” category includes all areas outside of population centers (i.e., areas with less than 1,000 people), which are collectively defined as rural areas (24). For the present analysis, place of residence was coded as 0 indicating “Small population center—1,000–29,999,” 1 indicating “Medium population center—30,000–99,999,” and 2 indicating “Large population center—100,000 or more.”

#### 2.3.2. Mental wellbeing

Self-reported mental health was used directly from the APS. Participants rated mental health on a five-point Likert-type scale, ranging from “Poor” to “Excellent.” Higher scores indicated positive perceived mental health status.

#### 2.3.3. Income

Income was assessed in the APS *via* the question, “Overall, in the past 12 months, was your household income enough to meet your household’s needs for transportation, housing, food, clothing, and other necessary expenses?” with options being “More than enough,” “Enough,” and “Not enough.” Items were reverse coded to reflect higher scores being associated with having enough income.

#### 2.3.4. Employment

Employment was assessed in the APS *via* a variable that identified if a person was employed or not employed during the reference week (i.e., the most recently completed seven-day period beginning on a Sunday and ending on the following Saturday). Options were “Employed” and “Unemployed,” and these were reverse coded with the higher score indicating being employed.

#### 2.3.5. Education

Education was assessed in the APS *via* a question about one’s highest level of education. Responses were grouped into the following categories: (1) Grade 8 or equivalent or lower; (2) Some secondary education; (3) Secondary school diploma or equivalent; (4) Some postsecondary education; (5) Postsecondary certificate or diploma below bachelor level; and (6) Bachelor decree or university certificate/diploma/degree above bachelor level. Higher scores indicated higher levels of educational attainment.

#### 2.3.6. Housing

Housing was assessed in the APS *via* the question, “How would you rate your level of satisfaction with your housing conditions?” with options being “Very satisfied,” “Satisfied,” “Neither satisfied nor dissatisfied,” and “Dissatisfied or very dissatisfied.” Response options were reverse coded with higher scores indicating greater satisfaction with living conditions.

### 2.4. Analytic procedure

The 2017 APS Public Use Microdata File (PUMF) was freely accessed online for this study. Consistent with the Tri-Council Policy Statement *Ethical Conduct for Research Involving Humans* (26), an approve request and documented exemption from the Lakehead University Research Ethics Board for use of secondary data was provided. Aligning with Statistics Canada regulations for use of the 2017 APS analytic file, all data were weighted by person to represent themselves as an individual case (i.e., person) as well as others within the population who were not sampled. Each individual case had a calculated weight that was used to calculate the number of people each individual case represented, which was based on intersecting population-level demographic characteristics (e.g., age, sex, Indigenous status, and region; (27)). Participant data with a response coded as “Valid skip,” “Do not know,” “Refusal,” or “Not stated” for any of our variables of interest was listwise deleted, resulting in initial analysis being completed with *n* = 8,168 respondents. Finally, aligning with Statistics Canada policies, cell counts were reviewed for any less than or equal to 10 individuals to correspond with respondent confidentiality protocols. No cell counts less than or equal to 10 were found for these analyses.

All statistical analyses were conducted using STATA. First, descriptive statistics were analyzed to collect frequency information. Second, bivariate associations between our social determinant of health variables were reported *via* Spearman rank correlations. Third, an ANOVA was used to determine the proportion of variance in self-reported mental health explained by the control variables only. Then, a second ANOVA was used to determine the proportion of variance in self-reported mental health explained by both control variables and our social determinant of health variables.

## 3. Results

### 3.1. Descriptive statistics

There were 8,168 First Nations individuals living off reserve whose responses were analyzed in this study. There were more female respondents than male respondents (55.7 vs. 44.3%, respectively). Compared to recent census data (28), females were slightly overrepresented within this sample. Respondents ranged in age from 15 to 55+, with the largest respondent age group being 55+ (31.5%) and the smallest respondent age group being 15–18 (4.3%). Proportions of age groups within this sample match those reported in recent census data (28), aside from a slight over representation of the age category “55 and over” and an under representation of individuals aged 14 or younger, who were not included in the APS 2017 survey. About half of respondents lived in a census metropolitan area (i.e., large population center; 49.7%), followed by those in other population centers (i.e., medium and small population center; 30.7%), followed by those living in rural areas (19.6%). Compared to census data, individuals living in rural areas were underrepresented within this sample (29). Additional sample characteristics regarding the social determinant of health variables (i.e., education, employment, income, and housing) for this sample is available in Table 1. Descriptive information for self-reported mental health was also calculated for the total sample (*M* = 2.54, *SD* = 0.02) and for each subgroup of each control and predictor variable included in our models (see Table 1).

### 3.2. Bivariate associations between predictor variables

Spearman rank correlations were run to assess the relationships between our predictor variables (i.e., income, employment, education, and housing). All correlations between variables were statistically significant, and all were positively correlated; however, the strengths of correlations varied. Income and housing were moderately correlated, while all other combinations of correlations were weakly correlated. Spearman rank correlation values and significance values are displayed in Table 2.

**Table 2:** Spearman rank correlations and significance values for predictor variables.

	Education	Employment	Income	Housing
Education	1.00			
Employment	0.26	1.00		
Income	0.17	0.21	1.00	
Housing	0.13	0.09	0.37	1.00

* All correlations presented here are significant (i.e., p = < 0.001).

### 3.3. Control variables ANOVA

First, an ANOVA was conducted with the control variables (i.e., age, sex, and place of residence). All control variables had significant main effects on self-reported mental health. Specifically, a significant main effect of sex indicated that men had higher self-reported mental health (*M* = 2.67) than women (*M* = 2.47), *F*(1, 8,159) = 107.29, *p* < 0.001, η^2^_p_ = 0.01. A significant main effect of age was also found, *F*(5, 8,159) = 10.95, *p* < 0.001, η^2^_p_ < 0.01, with increasing age bins corresponding with increasing self-reported mental health. A significant main effect of place of residence was also found, *F*(2, 8,159) = 7.71, *p* < 0.001, η^2^_p_ < 0.01, with increasing size of a population center corresponding with decreasing self-reported mental health. Overall, 2.03% of the variance in self-reported mental health was explained by age, sex, and place of residence variables. See Table 3 for additional details of this analysis.

**Table 3:** Predictors of self-reported mental health for First Nations individuals living off-reserve.

	Model 1: Control variables only					Model 2: Control and predictor variables				
Predictor	*F*	*p*	η^2^_p_	Lower 95% CI	Higher 95% CI	*F*	*p*	η^2^_p_	Lower 95% CI	Higher 95% CI
Age	10.95	<0.001	<0.01	0.003	0.010	18.49	<0.001	0.01	0.007	0.017
Sex	107.29	<0.001	0.01	0.009	0.018	79.71	<0.001	<0.01	0.006	0.014
Place of Residence	7.71	<0.001	<0.01	0.000	0.004	9.85	<0.001	<0.01	0.001	0.005
Education						5.54	<0.001	<0.01	0.001	0.006
Employment						50.80	<0.001	<0.01	0.003	0.010
Income						170.14	<0.001	0.04	0.032	0.049
Housing						81.15	<0.001	0.03	0.022	0.036
Constant	22.14	<0.001	0.02	0.014	0.027	74.38	<0.001	0.15	0.132	0.160
*R* ^2^_adjusted_	0.020					0.146				

### 3.4. Social determinants of health ANOVA

Next, a second ANOVA was conducted with both control variables (i.e., age, sex, and place of residence), as well as predictor variables (i.e., education, employment, income, and housing). All control and predictor variables had significant main effects on self-reported mental health. Again, there was a significant main effect of sex indicating that men had higher self-reported mental health (*M* = 2.67) than women (*M* = 2.47), *F*(1, 8,159) = 79.71, *p* = 0.001, η^2^_p_ = 0.01. A significant main effect of age was found again, *F*(5, 8,159) = 18.49, *p* < 0.001, η^2^_p_ = 0.01, with increasing age bins corresponding with increasing self-reported mental health. A significant main effect of place of residence was found again as well, *F*(2, 8,159) = 5.54, *p* < 0.001, η^2^_p_ < 0.01, with increasing size of a population center corresponding with decreasing self-reported mental health.

Examining the predictor variables of interest, a significant main effect of education was found, *F*(5, 8,159) = 9.85, *p* < 0.001, η^2^_p_ < 0.01, with greater educational attainment corresponding with increased self-reported mental health. A significant main effect of employment was also found, *F*(1, 8,159) = 50.80, *p* < 0.001, η^2^_p_ = 0.01, with employed respondents reporting higher self-reported mental health (*M* = 2.64) than unemployed respondents (*M* = 2.46). A significant main effect of income was found, *F*(2, 8,159) = 170.14, *p* < 0.001, η^2^_p_ = 0.04, with perceptions around having enough or more than enough income corresponding with increasing self-reported mental health. Simple effects tests revealed significant self-reported mental health differences across income conditions, with those indicating they had enough income reporting higher self-reported mental health (*M* = 2.60) than those indicating they did not have enough income (*M* = 2.17), *t*(6,341) = 14.33, *p* < 0.001, and with those indicating they had more than enough income reporting higher self-reported mental health (*M* = 2.85) than those indicating they had enough income (*M* = 2.60), *t*(6,264) = 8.33, *p* < 0.001. Finally, a significant main effect of housing was found, *F*(3, 8,159) = 81.15, *p* < 0.001, η^2^_p_ = 0.03, with increased satisfaction with housing corresponding with increased self-reported mental health. Overall, 14.58% of the variance in self-reported mental health was explained by these control and predictor variables, with the education, employment, income, and housing variables explaining an additional 12.55% of variance compared to the model with only control variables (i.e., age, sex, and place of residence). See Table 3 for additional details of this analysis.

## 4. Discussion

The purpose of the present study was to extend findings regarding CWB and individual mental wellbeing by examining the relationship between individual indicators of CWB (i.e., education, employment, income, and housing) and subjective mental health reports for First Nations respondents living off reserve *via* the data from a national, population level survey. Higher self-reported mental health was associated with individuals reporting having enough or more than enough income, being employed, having higher levels of educational attainment, and having greater satisfaction with their current housing conditions. These findings support existing models that describe how various social determinants of health can impact individual well-being, such as the CWB Index (9) and the *Social Determinants Model of Aboriginal Health* (3). These results also corroborate previous findings that linked the CWB total scores to other measures of subjective wellbeing (21).

Numerous studies have depicted how lower socio-economic status, including lower personal and household income, contributes to decreased mental health outcomes (30–32). The current study described how participants’ subjective reports of not having enough income were associated with decreased self-reported mental health among First Nations adults living off-reserve; to our knowledge, this is the first study of this kind to include this specific indicator of income instead of grouped income brackets. Subjective reports of having enough or not enough income provide the benefit of indicating whether participants feel they have enough income to meet their financial needs (e.g., rent, groceries) in various contexts (e.g., rising costs of rent, rising inflation), which cannot be obtained from income brackets alone. Our results are aligned with previous research with APS 2012 data from Indigenous individuals (First Nations, Inuit, and Métis) describing how falling in lower income categories (less than $40, 000) was affiliated with lower self-reported general health (33). Contrary to Bethune and colleagues (33), who found higher education was most strongly associated with better health outcomes, we found that perceptions of having sufficient income (i.e., enough or more than enough) were most strongly associated with higher mental health ratings.

One’s ability to meet their individual financial demands is associated with increased mental health outcomes (34). Preliminary analyses of an Ontario-based universal basic income program indicated those who received additional income reported improved mental health status (35). Similarly, Hajizadeh and colleagues (2021) demonstrated that if socio-economic status among Indigenous populations in Canada was bolstered to be similar to non-Indigenous, mean psychological distress scores and suicidal ideation/planning would decrease by over 25% (36). Given that there are bi-directional influences among social determinants of education, employment, income, and housing, assessing the singular effect of one determinant remains challenging, as factors are contextualized among one another. In large scale population studies, mediating one of these factors has affected the subsequent relationship of another factor on mental health outcomes, with a large emphasis placed on the mediating role of socioeconomic status (37–39).

Social disparities are inherently experienced differently across age, sex, and gender demographics. Although it was not possible to contextualize such differences across various gender categories due to a lack of data on gender, results of sex-based comparisons from the current study depicted that females were more likely to report lower mental health ratings. This is consistent with previous analyses of the APS and other studies of mental health of Indigenous individuals. Indigenous women are disproportionately exposed to stressors that can affect mental health outcomes, such as being likely to be single parents (40) or experience intimate partner violence (41) than their non-Indigenous or male counterparts. Analyses of a previous iteration of the APS have shown that when mental health outcomes were compared across sexes with consideration of income inequity, women reported significantly higher distress scores, while men had higher suicidal behaviors (36). With respect to age, studies with non-Indigenous populations have found that older adults tend to report fewer problems related to mental illness (42, 43). In line with these findings, analyses reported here indicated that increased age was predictive of increased self-reported mental health for First Nations individuals living off reserve.

Analyses showed that living in smaller communities was predictive of higher self-reported mental health. Although these results contrast previous literature depicting urban and rural differences in mental health outcomes within predominately non-Indigenous populations, these results align with some findings depicting wellness among Indigenous populations (33, 44). For example, although suicide attempts and deaths by suicide are higher in rural communities when examining population level data for individuals living in Ontario (36, 45), among Indigenous populations living off-reserve, those living in rural communities had statistically significant lower lifetime suicide ideation, attempts, and psychological distress compared to those living in larger communities (36). A further challenge remains extending these findings to be inclusive of all Indigenous communities, as the APS is designed solely for Indigenous individuals living off-reserve. With respect to deaths by suicide, living on-reserve (specifically in a remote community) is associated with significantly higher rates among First Nations populations, particularly among younger age groups (46). Differences with respect to self-reported mental health and social determinants across types of geographical locations are challenging to authentically contextualize within the current analyses, although these findings may be useful to inform future work regarding geographical differences in mental health outcomes. It is possible that geographical rurality may facilitate greater access to land-based activities, an ability to be more connected to one’s cultural through engagement in cultural practices or improve community belonging.

### 4.1. Study implications

Contrary to the findings presented here, interventions for those experiencing mental health concerns are largely individually-based (e.g., psychological or pharmacological vs. social interventions) and put the onus on individuals to apply tailored skills to mitigate one’s own mental health concerns (47). Emphasis is placed on one’s self-determination, autonomy, and motivation within treatment to apply provided strategies that can bolster mental health. Although such interventions are useful and will continue to be facilitated through individually-based psychological and counseling options, the clinical utility of such treatments may be overshadowed by one’s broader access to health promoting factors such as stable housing, employment, educational opportunities, or income stability (48). It can be challenging to engage in complex, introspective, or difficult behavioral-change processes when one’s basic needs are not consistently met, one must provide care to children or other dependents, or when one does not feel safe in their home or community environment. Similarly, when such individually-based mental health interventions fail due to increased barriers or poor living conditions created through inaccessibility to proximal or intermediate social determinants, onus is placed on the individual for this treatment failure, which can further exacerbate mental distress or reduce the likelihood of seeking similar services in the future.

Upstream interventions to address social determinants that can interfere with treatment or exacerbate mental health outcomes are required to authentically address mental health disparities experienced among First Nations populations. When such interventions are prioritized to be provided first, or even in conjunction with additional mental health treatments, they can facilitate increased engagement in individual mental health treatments, and potentially decrease likelihood of symptom exacerbation or recurrence. Research has demonstrated various ways in which each of the four social determinants of health can bolster mental well-being. In terms of housing stability, research examining Housing First options for First Nation youth have found that when youth are provided with safe and secure housing, without any requirements to engage in mental health services, they are more likely to seek these supports (49). Similarly, nutrition assistance programs, universal health care, universal basic income, and urban planning programs show increased mental health outcome for program users (35, 50). As well, access to education can also support increased access to school-based mental health programs and members of the community trained to respond to young people in crisis, which has been shown to be effective for reducing suicide rates in First Nations youth (51). Relevant mental health interventions may also require a shift to focus on, or simultaneously prioritize, not only the presenting mental health symptoms but also social determinants as a clinically and culturally-relevant focus of treatment.

### 4.2. Study limitations and future directions

The present research is not without limitations. First, this paper assessed only the four social determinants of health included in the CWB Index, and only mental wellbeing; in contrast, well-being can be shaped by many other variables and can be defined in many other ways. For example, the First Nations Mental Wellness Continuum Framework (FNMWCF) (52, 53) is rooted in culture and takes a more holistic approach when defining wellness and describing the factors that impact wellness. In the FNMWCF, there are four facets of mental wellness that correspond to the four quadrants of the medicine wheel (i.e., mental, physical, emotional, and spiritual wellbeing). However, only self-reported mental well-being was available in the APS 2017 data-set so self-reported physical, emotional, and spiritual wellbeing could not be assessed. In future iterations of the APS, questions regarding physical, emotional, and spiritual wellbeing should be included. However, examining mental well-being itself is still useful because it can come from increased hope, belonging, meaning, and purpose and because it intersects with the wellness of one’s family, community, society, and culture. Culture is highlighted as being at the center of mental wellness, implying that all health services and programs related to First Nations must go above and beyond creating culturally relevant programs by using culture as a starting point and then integrating current policies, strategies, and frameworks (52).

While study findings are not particularly novel, they provide corroboratory analyses using national, population-level data for theoretical frameworks such as those presented in Reading and Wein (3) regarding the associations between social determinants of health and mental well-being. To our knowledge, systematic reviews have mostly been used for assessing the individual impacts of various social determinants of health on mental well-being for First Nations individuals living in Canada (54, 55), and more broadly, for Indigenous individuals living in Canada (8, 56–59). We highlight the relationship between social determinants of health and mental wellbeing for First Nations individuals living off reserve using national, population-level data from the APS 2017, which fills a gap within the literature. Future research assessing First Nations wellness *via* large datasets like the APS 2017 should examine how accessing culture in various ways (e.g., *via* language, practices, ceremonies, knowledge, land, and values) (52) can impact a more holistic conceptualization of wellbeing (i.e., mental, physical, emotional, and spiritual wellbeing). Drawson and colleagues (60) also note that future research should consider other confounding factors when examining this relationship. This recommendation came after Drawson and colleagues (60) purposely demonstrated the importance of this statistically, using national data to show that, when examined in isolation, knowledge of traditional Indigenous language predicted reduced CWB scores, but when geographic remoteness was included as a predictor in the model, the relationship between knowledge of traditional language and CWB scores was no longer significant and instead geographic remoteness accounted for much of the variance. The former finding could have led to disastrous implications for funding or program planning if used alone (i.e., reduced funding for programs supporting the revitalization of traditional language use), and the latter finding demonstrates the necessity of contextualizing data interpretations (60). Additional recommendations for researchers using large-scale datasets from Indigenous communities can be found in Drawson and colleagues (60).

Another limitation of this research relates to additional gaps and challenges that exist within the APS survey. While the introduction of the APS was a significant improvement for addressing health related questions as it increased the quantity and quality of available Indigenous health data, there are still several issues that exist (3). For example, the APS includes First Nations individuals living off-reserve only, and First Nations individuals living on-reserve are accounted for *via* the First Nations Regional Longitudinal Health Survey (FNRLHS) (3). As different authorities are responsible for each survey, questions may be worded differently, and this makes it challenging to compare how the same concepts may differ for First Nations individuals living on- vs. off-reserve (3). Nelson and Wilson (57) also highlight that the broad categorization of Indigenous peoples into First Nations, Métis, and Inuit is a crude measure of identity that creates three homogeneous categories of Indigenous peoples and misses out on important variation. Because of this broad categorization, researchers are unable to point to specific nuances among groups or provide specific prevalence rates of mental illnesses for specific groups of First Nations peoples in Canada. Similarly, as the APS 2017 survey only reported binary responses for sex and do not capture gender, the results here cannot explicitly speak to the associations between social determinants of health and mental health for individuals whose gender does not correspond to their sex at birth (i.e., transgender men and transgender women) or responses from individuals who gender is not exclusively “man” or “women” (e.g., Two-Spirit individuals). Future iterations of the APS survey that allow for refined categorizations of gender will similarly allow for understanding and discussion of nuances between specific subgroups of First Nations peoples in Canada.

Future directions for assessing the relationship between First Nations well-being and various social determinants of health at a population level would include examining these concepts more holistically as outlined above. This could include models with strengths-based predictors, such as indicators of cultural connectedness (61), and models with broader well-being outcomes, such as those that assess mental, emotional, physical, and spiritual well-being (52, 53), and future iterations of the APS survey should assess each of these aspects of well-being. More complex statistical models could also be used, such as latent class analysis (62), which could be used to identify qualitatively different subgroups within populations (e.g., examining subgroups that have access to differing combinations of our social determinants of health variables, such as access to housing but not education, or access to income but not housing, and determining how these classes may be differentially associated with mental health outcomes). Other social determinants of health, such as presence of young children and accessibility to free childcare could also be included in future models. In addition, while the present results are cross-sectional (i.e., from many individuals at *one* time-point), if future models could be analyzed while including multiple iterations of the APS (i.e., longitudinal data points), such models could then be used to further support funding and public policy change for further access to additional resources (e.g., funding to support communities with increased access to cultural activities in addition to funding to support communities with better housing infrastructure). As well, using a community-based participatory framework (CBPR) to ensure that communities are involved in all stages of the research process involving the APS (from conception to interpretation to dissemination) could allow community members to define and conceptualize wellness and various social determinants in ways that are most meaningful for them (60). Incorporating these definitions and conceptualizations into the APS could then allow for collection of more accurate and valid data as well.

Future research could explore how changes to social determinants of health differentially affect mental health outcomes for First Nations individuals living off reserve when compared to other populations (e.g., First Nations individuals living on reserve, Métis populations, non-Indigenous populations, etc.). As comparisons of mental health outcomes between First Nations individuals living off reserve and living on reserve are difficult to obtain (likely due in part to the different authorities responsible for collecting these data as noted above, as well as limitations in data collection approaches with Indigenous people in general (63)), we present in this paragraph some literature showing differences in mental health outcomes for Indigenous vs. non-Indigenous populations after implementing changes directed at improving various social determinants of health. For example, while there are efforts to acknowledge and preserve Indigenous languages, knowledge, ceremonies, and traditional parenting practices in some schools, there are still instances that hinder positive relationships between school personnel and Indigenous families (e.g., reports to child welfare agencies causing family disruption) (64). These institutional practices continue to contribute to anxiety among Indigenous families in ways that are not experienced by non-Indigenous families, and can lead to poorer mental health outcomes (64). Similarly, while an urban Housing First initiative for Indigenous participants in Winnipeg, Canada contributed to a range of positive outcomes (e.g., environments of safety, self-control, and privacy), there were also significant structural constraints (e.g., a lack of culturally appropriate affordable housing; systemic erasure of Indigeneity from the urban sociocultural and political landscape of the city) (65) which could attenuate the mental health benefits in comparison to the mental health benefits that a non-Indigenous person might experience in an adjacent setting. A study summarizing the experiences of individuals experiencing houselessness also found that Indigenous participants described narrative identities filled with stories of racism, historical trauma, and disconnection from one’s culture(s), while those of non-Indigenous participants (specified in this study as White Canadian or European) (66) did not. These results indicated again that access to housing alone may not be enough to see similar improvements in mental health outcomes across populations—access to methods of cultural healing and reclamation will be required as well (66). Finally, one study found that gaps in suicide-related behaviors between Indigenous populations living off-reserve and non-Indigenous populations in Canada were largely due to differences in the effects of unobserved determinants (i.e., not socioeconomic factors such as income and employment which had been included in the analytical model) (67). The authors highlighted that variables they were unable to observe were impacting these differences in suicide-related behaviors (67); such differences could include factors such as systemic discrimination, and other systemic impacts stemming from colonization. These studies highlight that, while addressing the social determinants of health included in this paper are important, providing access to resources that support cultural resilience and advancing the process of Canadian reconciliation (e.g., addressing the 94 Calls to Action put forward by the Truth and Reconciliation Commission of Canada (68)) is needed to improve mental health outcomes for Indigenous people.

## 5. Conclusion

The current study has extended previous work, demonstrating that income security, housing satisfaction, higher education, and employment are associated with increased self-reported mental health among First Nations individuals living off-reserve. We have shown that individual perceptions of having enough or more than enough income are most strongly associated with increased mental health outcomes. The subjective nature of income reports is a novel contribution to existing literature, and depicts that is not necessarily one’s socioeconomic status, but also one’s perception of having basic needs met, is affiliated with increased mental health. Although future work can examine various interventions among social determinants of mental health experienced among First Nations communities, we hope the present findings can broaden understanding of existing disparities exist among First Nation communities by depicting the nature of these trends in a large-scale, population-based dataset. Addressing social determinants of mental health in an authentic way that centers Indigenous self-determination and knowledge is what will close these gaps. Careful consideration of how existing mental health services, and how subsequent health interventions to address these social determinants contribute to quantifiable reductions in mental health outcomes is required. Until this occurs, we will continue to be engaged in crisis-focused, symptom-based, or downstream approaches that will not fully address factors associated with decreased mental wellbeing.

## Data availability statement

Publicly available datasets were analyzed in this study. This data can be found at: https://abacus.library.ubc.ca/dataset.xhtml?persistentId=hdl:11272.1/AB2/U0UXQJ.

## Ethics statement

Consistent with the Government of Canada’s Tri-Council Policy Statement “Ethical Conduct for Research Involving Humans”, an approve request and documented exemption from the Lakehead University Research Ethics Board for use of secondary data was provided. Before the start of the 2017 APS survey, participants received introductory letters explaining the purpose of the survey and provided informed consent to participate in that survey.

## Author contributions

ET, LH, and CM: conceptualization, interpretation, and writing—review and editing. ET and LH: methodology and writing—original draft preparation. LH: analysis. ET and CM: supervision. All authors contributed to the article and approved the submitted version.

## Funding

LH’s participation in this project was supported by a Canadian Institutes of Health Research Canadian Graduate Scholarship (Master’s Level; CIHR PIN: 96509). CM’s participation in this project was partially supported by the Canada Research Chairs Program (grant number: CRC-2019-00069) and the Canada Foundation for Innovation (grant number: 33721). ET’s participation was supported by a Canadian Institutes of Health Research Banting Postdoctoral Fellowship (application number: 454119).

## Conflict of interest

The authors declare that the research was conducted in the absence of any commercial or financial relationships that could be construed as a potential conflict of interest.

## Publisher’s note

All claims expressed in this article are solely those of the authors and do not necessarily represent those of their affiliated organizations, or those of the publisher, the editors and the reviewers. Any product that may be evaluated in this article, or claim that may be made by its manufacturer, is not guaranteed or endorsed by the publisher.

## Footnotes

^1^The terminology used to describe Indigenous populations matches the terminology referenced in the literature cited. Various terms cannot be used interchangeably. For example, the term "Indigenous" in Canada represents distinct groups of people including First Nations, Inuit, and Métis populations.
